# The prevalence of serious bacterial infections in infants 90 days and younger with viral respiratory tract infections

**DOI:** 10.15537/smj.2022.43.9.20220400

**Published:** 2022-09

**Authors:** Abdullah I. Almojali, Musab S. Alshareef, Othman F. Aljadoa, Fahad F. Alotaibi, Emad M. Masuadi, Tahir K. Hameed

**Affiliations:** *From the Department of Pediatrics (Almojali, Alshareef, Aljadoa, Alotaibi, Hameed), King Abdullah Specialized Children’s Hospital, King Abdulaziz Medical City; from King Abdullah International Medical Research Center (Almojali, Alshareef, Aljadoa, Alotaibi, Masuadi, Hameed), Ministry of National Guard - Health Affairs; and from the College of Medicine (Almojali, Alshareef, Aljadoa, Alotaibi, Masuadi, Hameed), King Saud Bin Abdulaziz University for Health Sciences, Riyadh, Kingdom of Saudi Arabia.*

**Keywords:** respiratory tract infections, infant, newborn, urinary tract infections, neonatal sepsis, bacteremia

## Abstract

**Objectives:**

To determine the prevalence and risk factors of serious bacterial infections (SBIs) in infants 90 days and younger with a confirmed respiratory tract infection (RTI).

**Methods:**

A retrospective cross-sectional study was carried out of infants 90 days and younger who were admitted to King Abdullah Specialized Children’s Hospital, Riyadh, Saudi Arabia, from January 2019 to December 2020, with polymerase chain reaction (PCR)-proven RTI. Cultures from the urine, blood, and cerebrospinal fluid were reviewed with the patients’ demographic information and clinical presentation.

**Results:**

Of 322 patients with a viral RTI, 21 (6.5%) had a concurrent urinary tract infection (UTI), and no patients had bacteremia or bacterial meningitis. The risk of a concurrent SBI was 4 times higher in neonates (odds ratio [OR]=4.66, 95% confidence interval [CI]: [1.32-16.47]). Previously healthy infants were at lower risk to have a SBI in comparison to those with chronic diseases or renal abnormalities (OR=0.23, 95% CI: [0.09-0.61]). In addition, male gender (OR=3.49, 95% CI: [1.07-11.38]) and abnormal urinalysis (OR=4.12, 95% CI: [1.48-11.42]) were predictors of SBIs. There was no statistically significant association between the number or type of detected viruses and SBIs.

**Conclusion:**

No cases of invasive bacterial infections were found in infants with PCR-proven viral RTIs. There is a risk of having a concurrent UTI in this cohort of patients. Neonates had a higher risk of UTIs as compared to older infants.

Viral respiratory tract infections (RTIs) are the most frequent cause of emergency department visits in the pediatric population.^1^ Internationally, respiratory syncytial virus (RSV), human metapneumovirus, adenovirus, and human rhinovirus are the most frequent causes of RTIs in children.^2^ Respiratory tract infections are usually self-limiting and clinically diagnosed with no need for extensive investigations. However, in neonates (0-28 days of age) and young infants (29-90 days of age), there is a concern of concurrent serious bacterial infections (SBIs) which prompts many practitioners to do a full septic work-up, initiate antibiotics, and admit this group of patients.^3,4^

Serious bacterial infections has traditionally been defined as a urinary tract infection (UTI), bacteremia, or bacterial meningitis. Literature indicates that the risk of SBI in neonates with RTI is not negligible, and appears to be lower in young infants 29-90 days of age compared with neonates.^5,6^ Urinary tract infections are the most common SBI occurring concurrently with viral RTIs in infants 90 days of age and younger. In a systematic review of occult SBI in young infants with bronchiolitis, the overall prevalence of UTI was found to be 3.3%.^7^ Another study of 4778 infants less than 60 days of age found that the rate of specific SBIs in virus-positive patients is lower compared to patients with no proven respiratory infection.^8^

Although some guidelines stratify and guide the management of febrile young infants, including those with proven viral infections, these guidelines are still controversial and debated.^9-12^ Recent guidelines from the American Academy of Pediatrics stated that the risk of invasive bacterial infections (IBIs) appears to be less in young infants with a positive viral respiratory test result. However, more studies are needed to give guidance whether less work-up should be carried out in the febrile young infant if there is a positive viral test.^13^ Currently, in Saudi Arabia, as part of the routine immunization schedule, the pneumococcal conjugate and hemophilus influenzae type B vaccines are given at 2 months of age to prevent IBIs from these bacteria.

Few studies have investigated the prevalence of viral RTIs in pediatric patients in Saudi Arabia. Respiratory syncytial virus was the most frequent etiology in one study and rhinovirus in another.^14,15^ An observational study found that 58% of febrile infants under 90 days of age who presented at an emergency department without an apparent source of infection had a positive polymerase chain reaction (PCR) for respiratory viruses.^16^ However, these studies did not explore the risk of concurrent SBIs in children with viral RTIs.

The aim of our study was to determine the prevalence and risk factors of a SBI in infants 90 days of age and younger with a proven RTI. Increased understanding of the prevalence and the risk factors may decrease the use of invasive testing, antibiotic use, and ultimately improve patient care and quality.

## Methods

A retrospective cross-sectional study was carried out at King Abdullah Specialist Children’s Hospital, Ministry of National Guard-Health Affairs, Riyadh, Saudi Arabia. From January 2019 to December 2020, 816 infants 90 days of age and younger were admitted to the hospital with a confirmed viral RTI (one or more viruses found on a respiratory multiplex PCR taken from a nasopharyngeal aspirate). They were divided in 3 main age groups: 0-28 days (n=176), 29-60 days (n=341), and 61-90 days of age (n=299). Patients in each age group were sub-divided in 4 groups based on gender and admission year, creating 12 groups. Infants that were born at term and with no chronic diseases (such as chronic lung disease, immunodeficiency, or genetic disorders) or renal abnormalities (such as hydronephrosis or vesicoureteral reflux) were labeled as previously healthy. Respiratory samples collected after 7 days from the date of presentation were excluded from the study, as these samples were considered nosocomial infections. Viruses detected more than once during a single clinical episode occurring within a 4-week period was counted once.

A serious bacterial infection was defined as a UTI, bacteremia, or bacterial meningitis. Urinary tract infection was defined as a positive urine culture of a bacterial uropathogen of >10,000 cfu/mL in an appropriately obtained specimen (catheter or suprapubic aspirate). Patients with a positive urine culture obtained from a bag collection were excluded. Urinalysis with positive nitrites, leukocyte esterase, or ≥5 WBC/hpf was labeled as abnormal. For bacteremia and bacterial meningitis, we considered the isolation of a pathogenic species as a true infection. Isolation of common skin flora (such as, *Staphylococcus epidermidis*) were considered as contaminants. To obtain a confidence interval (CI) of 95% and a 5% margin of error, a sample size of 280 patients was required. An additional 15% was added to the sample size to ensure adequacy of the data and to compensate for the excluded patients, resulting in a sample of 322 patients. To obtain an unbiased sample size representing the whole population, participants were selected from the 12 groups through stratified random sampling, using Microsoft Excel 2019. The study was carried out in adherence with ethical principles. It was carried out after obtaining approval from the Institutional Review Board of King Abdullah International Medical Research Center, Riyadh, Saudi Arabia.

### Statistical analysis

The data was entered in Microsoft Excel 2019 and analyzed with the Statistical Package for the Social Sciences, version 23 (IBM Corp., Armonk, NY, USA). The categorical variables are presented as frequency and percentage, and the continuous variables as a mean and standard deviation (SD). The association between SBI (UTI, bacteremia, and meningitis) with PCR-proven RTI as well as the demographic variables were examined using the Pearson’s Chi-square test. Serious bacterial infection was used as a dependent variable, and RTI and the demographic variables as independent variables. A test with a *p*-value of <0.05 was considered significant.

## Results

Of the 322 patients, there were 179 (55.6%) males and 143 (44.4%) females. For the age distribution, 90 (28%) patients were neonates, 125 (38.8%) 29-60 days of age, and 107 (33.2%) 61-90 days of age. The majority of the study sample were previously healthy (77.9%). Subjective or objective fever was present in 246 (76.4%) patients. The other demographic data and clinical characteristics are shown in Table 1.

**Table 1:** - Demographic data and clinical characteristics of the sample (N=322).

Characteristic	n (%)
***Gender***	
Male	179 (55.6)
Female	143 (44.4)
***Age group***	
0-28 days	90 (28.0)
29-59 days	125 (38.8)
60-90 days	107 (33.2)
***Gestational age***	
Preterm	45 (14.0)
Term	277 (86.0)
***Year***	
2019	217 (67.4)
2020	105 (32.6)
***Previously healthy***	
No	71 (22.1)
Yes	250 (77.9)
***Febrile***	
No	76 (23.6)
Yes	246 (76.4)
***Hypoactivity***	
No	221 (68.6)
Yes	101 (31.4)
***General appearance***	
Well	284 (88.2)
Ill	38 (11.8)

Values are presented as a number and percentage (%).

Of all the positive nasopharyngeal aspirates, RSV (49.1%) was the most frequent virus, followed by human rhinovirus/enterovirus (47.5%). Single viral infections were detected in 82.3% and 17.7% had more than one detected virus. Blood and urine samples were sent for culture for most of the patients (92%) and cerebrospinal fluid (CSF) cultures for 47.2%.

Of the 322 patients with proven viral RTIs, 21 (6.5%) patients had a UTI. No patient had true bacteremia or bacterial meningitis. However, 11 (3.4%) patients had contaminated blood cultures. Of the 21 patients with an UTI, the most frequently isolated uropathogens were *Escherichia coli* (61.9%), followed by *Klebsiella several species* (23.8%). Viral meningitis was detected in 10 (3.1%) patients, most frequently with enterovirus and followed by human herpesvirus 6 (n=3). All the patients who had enterovirus meningitis had a positive respiratory PCR for human rhinovirus/enterovirus (Table 2).

**Table 2:** - Respiratory viral infections and serious bacterial infections.

Variable		n (%)
***Viruses isolated***		
Single virus isolated		265 (82.3)
Multiple viruses isolated		57 (17.7)
***Respiratory multiplex PCR result***		
RSV	158 (49.1)	
Human rhinovirus/enterovirus	153 (47.5)	
Influenza A/B	15 (4.7)	
Corona HKU, 229E, NL63, and OC43	14 (4.4)	
Adenovirus	12 (3.8)	
Parainfluenza virus 1,2,3, and 4	10 (3.1)	
***Bordetella Pertussis***	8 (2.5)	
Human metapneumovirus	6 (1.9)	
SARS-CoV-2	6 (1.9)	
***Urine culture***		
No growth	269 (83.5)	
Not done	30 (9.3)	
*Escherichia coli*	13 (4.0)	
*Klebsiella several species*	5 (1.6)	
Candida	2 (0.6)	
*Staphylococcus aureus*	1 (0.3)	
*Enterobacter cloacae*	1 (0.3)	
*Citrobacter koseri*	1 (0.3)	
***Blood culture***		
No growth	285 (88.5)	
Not carried out	26 (8.1)	
Staphylococcus hominis*	7 (2.2)	
Staphylococcus epidermidis*	3 (0.9)	
Staphylococcus salivarius*	1 (0.3)	
***CSF PCR/culture results***		
Not carried out	143 (44.4)	
Negative	141 (43.8)	
Refused	19 (6.0)	
Failed	8 (2.5)	
Enterovirus	7 (2.2)	
Human herpesvirus 6	3 (0.9)	
Staphylococcus epidermidis*	1 (0.3)	

Values are presented as a number and percentage (%). *Contaminated samples. PCR: polymerase chain reaction, RSV: respiratory syncytial virus, SARS-CoV-2: severe acute respiratory syndrome coronavirus 2, CSF: cerebrospinal fluid

As shown in Table 3, just over half of the patients who developed a SBI were neonates with statistically significant association (*p*=0.034). Male gender was significantly associated with SBIs (*p*=0.016). The proportion of SBIs was significantly higher in patients who were not previously healthy (*p*<0.001). No significant association was found between SBI and the number or type of detected viruses. Logistic regression was carried out to identify the predictors of SBI (the dependent variable) as shown in Table 4. The risk of a concurrent SBI is 4 times higher in neonates (odds ratio [OR]=4.66, 95% CI: [1.32-16.47]). Previously healthy infants were at lower risk to have a SBI in comparison to those with chronic diseases or renal abnormalities (OR=0.23, 95% CI: [0.09-0.61]). In addition, male gender (OR=3.49, 95% CI: [1.07-11.38]) and abnormal urinalysis (OR=4.12, 95% CI: [1.48-11.42]) were predictors of SBIs.

**Table 3:** - Risk of serious bacterial infections in patients with proven viral respiratory tract infection.

Characteristic	Viral RTI	SBI	*P*-values
***Age group***			
0-28 days	79 (87.8)	11 (12.2)	0.034*
29-59 days	119 (95.2)	6 (4.8)	
60-90 days	103 (96.3)	4 (3.7)	
***Gender***			
Male	162 (90.5)	17 (9.5)	0.016*
Female	139 (97.2)	4 (2.8)	
***Previously healthy***			
Yes	240 (96.0)	10 (4.0)	0.002*
No	60 (84.5)	11 (15.5)	
***Febrile***			
No	74 (97.4)	2 (2.6)	0.083
Yes	227 (92.3)	19 (7.7)	
***Hypoactivity***			
No	207 (93.7)	14 (6.3)	0.841
Yes	94 (93.1)	7 (6.9)	
***Blood WBC***			
5000-15000	221 (93.2)	16 (6.8)	0.781
<5000 or >15000	80 (94.1)	5 (5.9)	
***Urinalysis/dipstick***			
Normal	205 (96.7)	7 (3.3)	<0.001*
Not carried out	28 (96.6)	1 (3.4)	
Abnormal^†^	68 (84.0)	13 (16.0)	
***Viruses isolated***			
Single	249 (94.0)	16 (6.0)	0.300
Multiple	52 (91.2)	5 (8.8)	
***RSV***			
Negative	154 (93.9)	10 (6.1)	0.753
Positive	147 (93.0)	11 (7.0)	
***Rhinovirus/enterovirus***			
Negative	159 (94.1)	10 (5.9)	0.644
Positive	142 (92.8)	11 (7.2)	

Values are presented as a number and percentage (%). *P*-values were calculated using the Pearson’s Chi-square test or Fisher’s exact test. *Significant *p*-value at <0.05). ^†^With positive nitrites, leukocyte esterase, or ≥5 WBC/hpf. RTI: respiratory tract infection, SBI: serious bacterial infection, WBC: white blood cells, RSV: respiratory syncytial virus

**Table 4:** - Predictors of serious bacterial infections in patients with proven viral respiratory tract infection.

Characteristics	*P*-value	OR	95% CI	
			Lower	Upper
***Age group***				
0-28 days	0.017	4.66	1.32	16.47
29-59 days	0.656	1.36	0.35	5.28
60-90 days*		1.00		
***Gender***				
Male	0.038	3.49	1.07	11.38
Female*		1.00		
***Previously healthy***				
Yes	0.003	0.23	0.09	0.61
No*		1.00		
***Urinalysis***				
Abnormal	0.007	4.12	1.48	11.42
Not carried out	0.957	1.06	0.12	9.27
Normal*		1.00		

*Reference group. OR: odds ratio, CI: confidence interval

## Discussion

The present study explored the risk of serious bacterial infections with PCR- confirmed viral respiratory tract infection in infants 90 days old and younger. Our main findings were that the overall risk is low, with UTIs being the only SBIs detected. The vast majority of infants that had UTIs were febrile. There were no cases of bacteremia or bacterial meningitis in our cohort of patients. Additionally, young infants with a confirmed viral infection have low risk of invasive bacterial infections and this may help limit investigations and improve antibiotic stewardship.

Of the patients with a proven viral RTI, just over 6% had a concurrent SBI and all of these were UTIs. The overall risk of bacteremia and bacterial meningitis in febrile infants (<90 days) is less than 2% and 0.5%.^12^ Studies have confirmed that the risk of concurrent SBIs in infants with proven viral RTIs is lower than in infants without viral RTIs.^7,8,12,13,17,18^ The findings of our study are in line with other research as no cases with bacteremia or bacterial meningitis were found, and 6.5% of the patients had a UTI.

The risk of UTIs in febrile infants less than 90 days without a source is estimated to be 10%.^8,13,19^ However, this risk is found to be lower in patients with viral RTIs, estimated to be 1.9-5.7%.^7,18,19^ Similar to these studies, we found a risk of UTI (6.5%) in young infants with documented viral RTIs. If we applied a stricter definition of UTI (such as, positive urine culture with pyuria), the risk of UTI would be lower (4.0%). Our study found that the younger age group (0-28 days of age) were more likely to have UTIs as compared to infants 29-90 days of age. This is consistent with studies on SBIs, including UTIs, where there is a higher risk in neonates compared to young infants.^5,6^ The young infants in our study with a UTI were almost all febrile and the majority had a urinalysis suggestive of a UTI. As recommended in the new Clinical Practice Guidelines by the American Academy of Pediatrics on the evaluation of well-appearing young febrile infants, urinalysis is a key predictor to identify the infants with a greater risk of UTI.^13^ Male gender was strongly associated with UTI in our study and this is a known risk factor for UTIs in young infants.^20^ This risk is markedly influenced by the circumcision status, but unfortunately it could not be evaluated in our study due to lack of documentation in the medical records.^20^

A study carried out at Texas Children’s Hospital, Texas, United States of America, proposed a possible association between the type of the virus and the risk of SBI. It was found that having mucosa-restricted viruses such as RSV and influenza virus is associated with a lower risk of SBI than a systemic virus such as adenovirus or enterovirus.^16^ However, we could not find an association between the type of the virus and the risk of SBI. In addition, we had 6 patients with SARS-CoV-2 (COVID-19). None of these patients had an SBI. While our number of COVID-19 patients was small in this cohort, recent evidence suggests that there is no association between the risk of concurrent SBI or the severity of respiratory illness with this novel virus.^21,22^

Although we did not observe any cases of true bacteremia, 11 (3.4%) patients had contaminated blood cultures. This is a very prevalent issue as 63-88% of positive blood culture obtained from infants less than 90 days old are contaminated.^23-25^ This could be a burden for the family and the health care system as it may result in unnecessary admission, investigations, use of antibiotics, and prolong the length of stay.^12^

### Study limitations

The study was carried out in one tertiary care center, and thus results cannot be generalized. In addition, while our finding of the risk of SBI in young infants with a viral RTI is consistent with other studies, we did not compare the frequency of SBI in young infants without documented viral pathogens. Also, CSF samples were not obtained in almost half of our subjects as bacterial meningitis was not suspected based on their clinical presentation. Nevertheless, as this study was carried out on admitted patients, we made sure that all those patients whose CSF was not obtained did not deteriorate during their hospital stay and that they were discharged home with a final diagnosis of viral RTIs.

In conclusion, neonates and young infants 90 days of age and younger with PCR-proven viral RTIs have a generally low risk of a concurrent SBI. Urinary tract infections are still common in young infants with RTIs, and it would be prudent to send a urinalysis and urine culture in febrile young infants in this population. There were no cases of invasive bacterial infections found in our cohort of patients, even those that were febrile. Further studies with larger numbers of patients are needed to determine if there is indeed a different SBI risk with different viruses and to explore risk factors that make some young infants with viral RTI more susceptible to UTIs.
